# Efficacy of Neuroendoscopic Treatment for Septated Chronic Subdural Hematoma

**DOI:** 10.3389/fneur.2021.765109

**Published:** 2022-01-11

**Authors:** Jianhong Deng, Fangyu Wang, Haojie Wang, Mingpei Zhao, Guorong Chen, Huangcheng Shangguan, Lianghong Yu, Changzhen Jiang, Wenhua Fang, Peisen Yao, Dezhi Kang, Shufa Zheng

**Affiliations:** ^1^Department of Neurosurgery, Guangze County Hospital, Nanping, China; ^2^Department of Neurosurgery, Neurosurgery Research Institute, The First Affiliated Hospital, Fujian Medical University, Fuzhou, China; ^3^Fujian Key Laboratory of Precision Medicine for Cancer, The First Affiliated Hospital, Fujian Medical University, Fuzhou, China; ^4^Key Laboratory of Radiation Biology of Fujian Higher Education Institutions, The First Affiliated Hospital, Fujian Medical University, Fuzhou, China

**Keywords:** septated chronic subdural hematoma, neuroendoscopic treatment, craniotomy, safely and effectively, minimally invasive neurosurgery

## Abstract

**Objective:** Neuroendoscopic treatment is an alternative therapeutic strategy for the treatment of septate chronic subdural hematoma (sCSDH). However, the safety and efficacy of this strategy remain controversial. We compared the clinical outcomes of neuroendoscopic treatment with those of standard (large bone flap) craniotomy for sCSDH reported in our center. Furthermore, the safety and efficacy of the neuroendoscopic treatment procedure for sCSDH were evaluated.

**Methods:** We retrospectively collected the clinical data of 43 patients (37 men and six women) with sCSDH who underwent either neuroendoscopic treatment or standard (large bone flap) craniotomy, such as sex, age, smoking, drinking, medical history, use of antiplatelet drugs, postoperative complications, sCSDH recurrence, length of hospital stay, and postoperative hospital stay. We recorded the surgical procedures and the neurological function recovery prior to surgery and 6 months following the surgical treatment.

**Results:** The enrolled patients were categorized into neuroendoscopic treatment (*n* = 23) and standard (large bone flap) craniotomy (*n* = 20) groups. There were no differences in sex, age, smoking, drinking, medical history, antiplatelet drug use, postoperative complications, and sCSDH recurrence between the two groups (*p* > 0.05). However, the patients in neuroendoscopic treatment group had a shorter length of total hospital stay and postoperative hospital stay as compared with the standard craniotomy group (total hospital stay: 5.26 ± 1.89 vs. 8.15 ± 1.04 days, *p* < 0.001; postoperative hospital stay: 4.47 ± 1.95 vs. 7.96 ± 0.97 days, *p* < 0.001). The imaging and Modified Rankin Scale at the 6-month follow-up were satisfactory, and no sCSDH recurrence was reported in the two groups.

**Conclusions:** The findings of this study indicate that neuroendoscopic treatment is safe and effective for sCSDH; it is minimally invasive and could be clinically utilized.

## Introduction

Chronic subdural hematoma (CSDH) refers to subdural hemorrhage, which usually occurs 3 weeks after traumatic brain injury (1). However, the pathogenesis of CSDH has not been fully elucidated. If the hematoma is surrounded by an envelope, repeated bleeding can occur. A pseudomembrane or fibrous septum in the hematoma cavity can form septations in the hematoma. This can lead to the formation of a septated CSDH (sCSDH) with septate cavities. Four methods are commonly used to treat CSDH: conservative (medical) treatment, twist-drill craniostomy, burr hole evacuation, and large bone flap craniotomy (2, 3).

For CSDH, treatment with atorvastatin combined with low-dose dexamethasone achieved favorable clinical results (2). However, Hutchinson et al. reported that dexamethasone was not beneficial in the conservative treatment of CSDH due to more adverse events than placebo (4). For non-septated CSDH, twist-drill craniostomy, and burr hole evacuation may be the optimal treatment options (5, 6); however, treatment of sCSDH remains a therapeutic challenge. Treatment with twist-drill craniostomy, burr hole evacuation, or irrigation with saline during and after the surgery can fail to achieve the satisfactory clinical results due to the septation of the hematoma by a fibrous septum, which prevents outflow of the hematoma fluid. Therefore, unsatisfactory sCSDH outcomes are often associated with hematoma recurrence or residual hematoma and can require a further large bone flap craniotomy (7). Although patients with sCSDH treated with large bone flap craniotomy are reported to have relatively favorable outcomes (7), it is a massively traumatic neurosurgery that results in large wounds, high infection rates, and high incidence rate of epilepsy (8).

Advances in microscopy and neuroendoscopy technology allow sCSDHs to be removed effectively by neuroendoscopic treatment (9). Currently, the neuroendoscopic treatment is considered the conventional treatment for CSDH. However, the advantages and disadvantages of performing neuroendoscopic treatment in the treatment of sCSDH, as compared with large bone flap craniotomy, are rarely reported. Therefore, this study retrospectively collected the clinical records of 43 patients with sCSDH treated with either neuroendoscopic treatment or standard (large bone flap) craniotomy in our center, and the clinical data and characteristics were compared and analyzed.

## Materials and Methods

### Ethics Statement

The Ethics Committee of the First Affiliated Hospital of Fujian Medical University, Fujian, China, approved the study and waived the requirement for written informed consent (the number/ID of the ethics approval: MRCTA, ECFAH of FMU [2019] 123).

### General Information

This study retrospectively collected the clinical data of 43 patients with sCSDH who underwent neuroendoscopic treatment or standard (large bone flap) craniotomy, such as sex, age, smoking, drinking, medical history, use of antiplatelet drugs, postoperative complications, sCSDH recurrence, length of hospital stay, and postoperative hospital stay. The surgical procedures, and neurological function recovery after surgery and 6 months following surgical treatment, were recorded. The diagnoses of sCSDH were based on imaging using head CT or MRI, clinical manifestations, and the medical history of patient. The thickness of the subdural hematoma in patients with sCSDH needed to be >2 cm to receive neuroendoscopic treatment.

### Surgical Procedure

Following the diagnosis of sCSDH, if there were no obvious surgical contraindications, the CSDH was evacuated under general anesthesia. In a standard (large bone flap) craniotomy, a question mark–shaped skin incision was made, and a fronto-temporoparietal bone flap procedure was performed. The subdural hematoma was evacuated, and an outer membranectomy was performed following the incision of the dura mater. However, an inner membranectomy was not performed to avoid injury to the cortical brain surface. For a more detailed craniotomy protocol, please refer to the surgical procedure for refractory CSDH by Matsumoto et al. (10).

**Figure 1:**
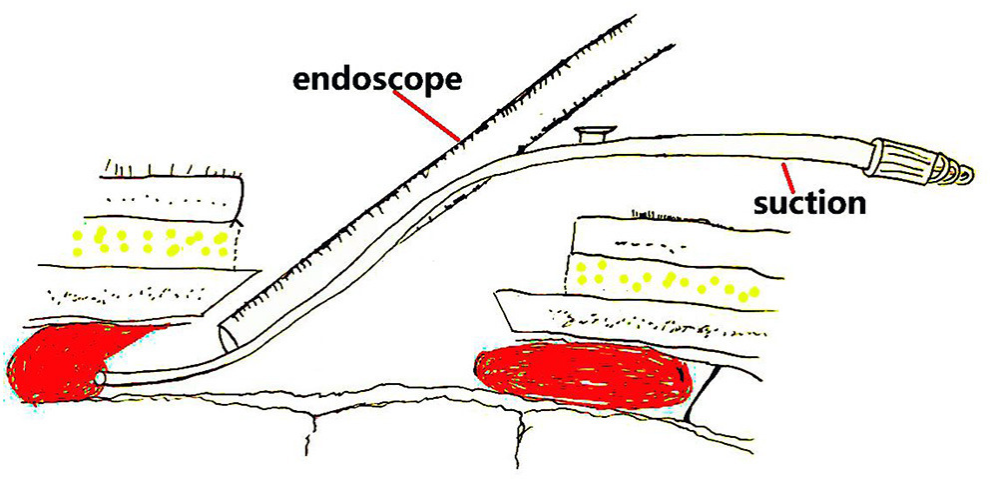
The relationship between the endoscope, suction device, and bone window.

**Figure 2:**
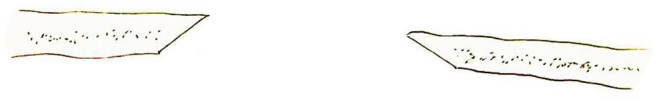
The flask-shaped bone window for neuroendoscopic removal of the subdural hematoma.

**Figure 3:**
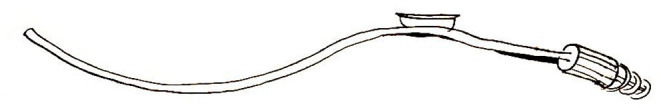
Suction device with an S-shaped curve for neuroendoscopic removal of the subdural hematoma.

**Figure 4:**
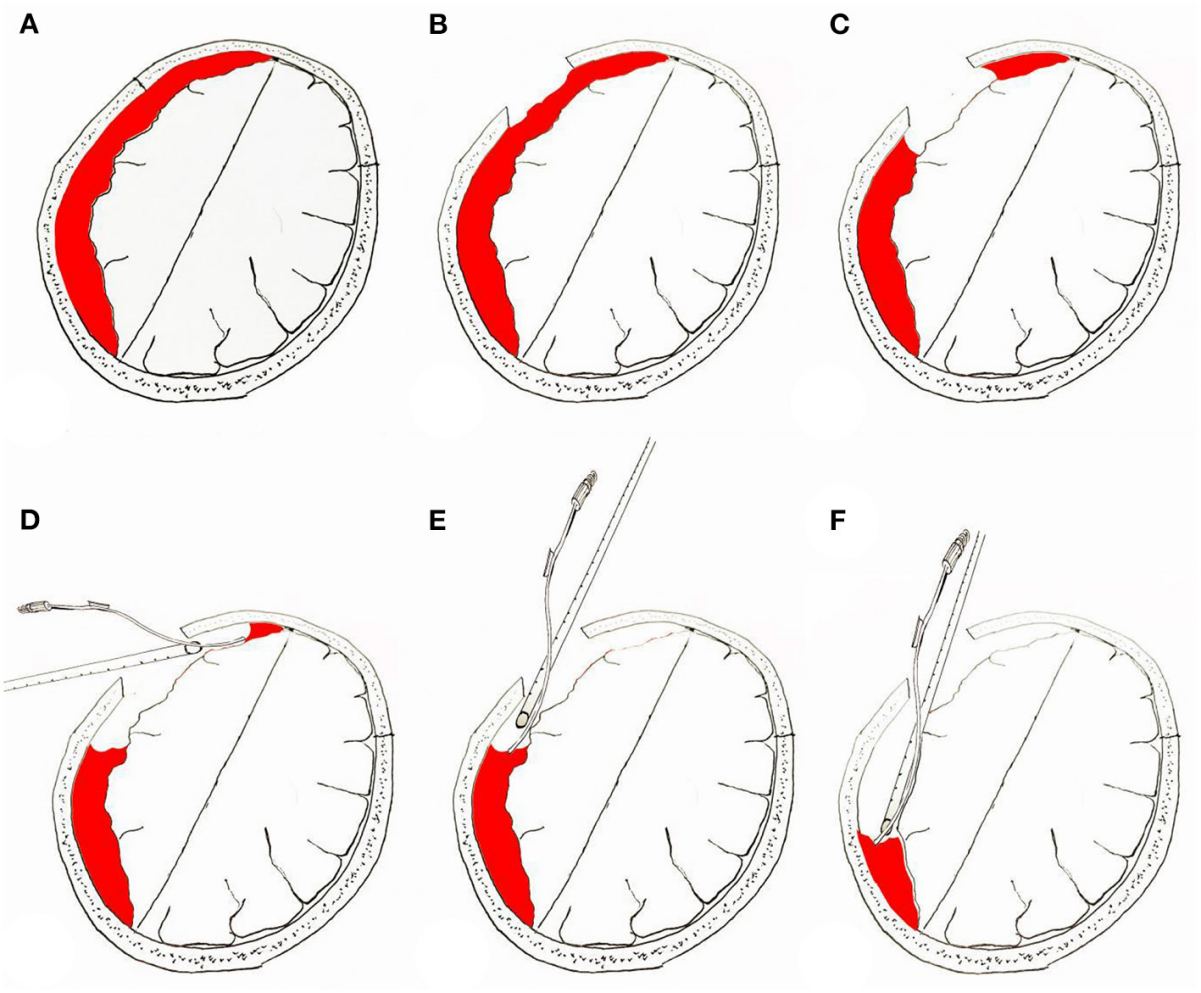
Surgical procedure for neuroendoscopic removal of septated chronic subdural hematoma. **(A)** Prior to neuroendoscopic removal of chronic subdural hematoma. **(B)** Location of bone window in the skull. **(C)** Removal of the hematoma under the bone window to make space for the neuroendoscope to enter the subdural space. **(D–F)** Hematoma clearance under neuroendoscopy; the “push” effect of the suction device is used to remove the hematoma distant from the bone window.

**Figure 5:**
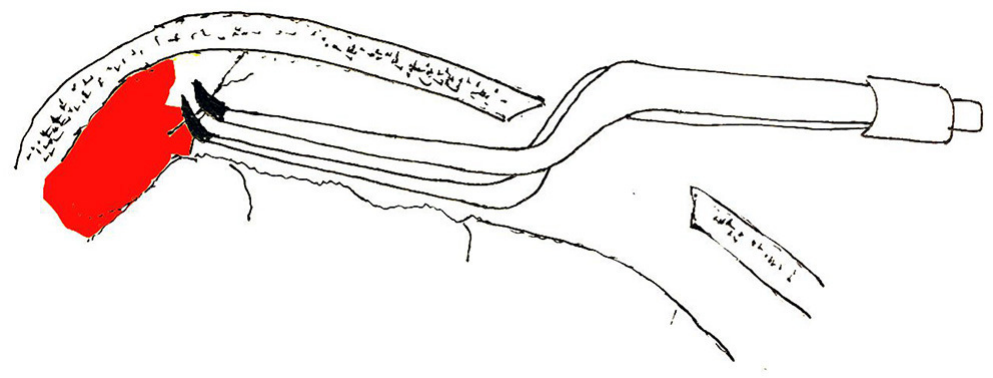
The tip of the bipolar forceps is bent backward to facilitate subdural hemostasis.

**Figure 6:**
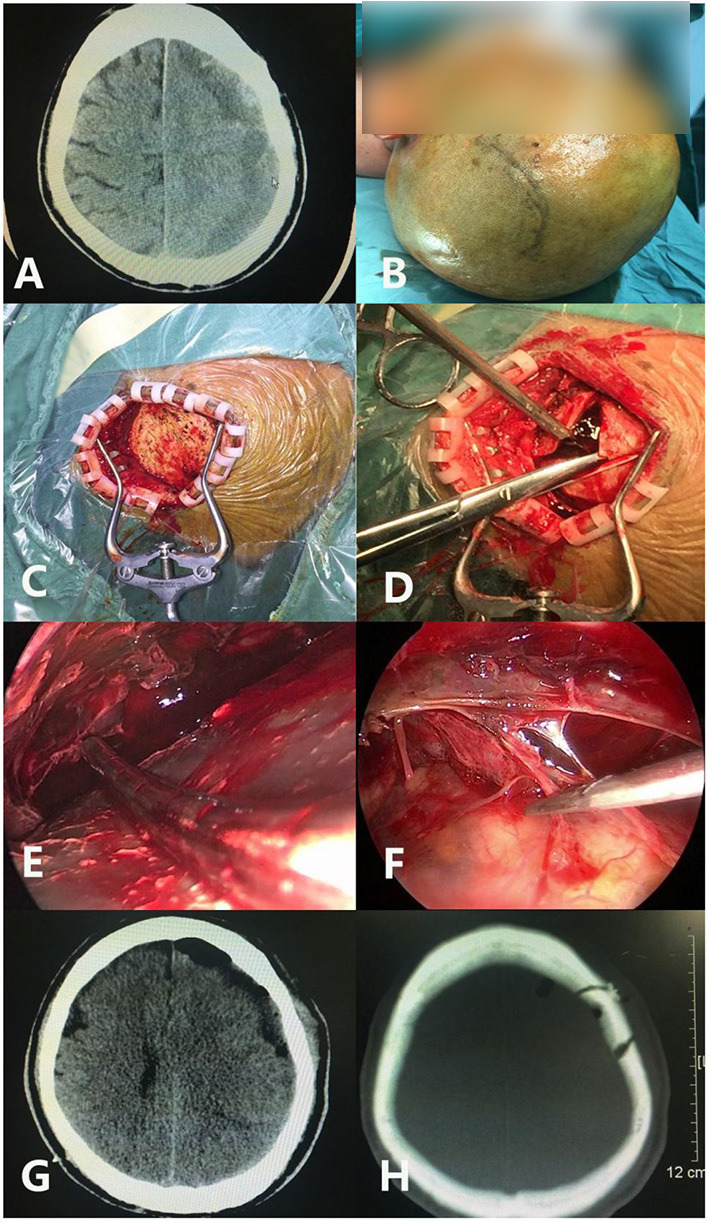
A case of septated chronic subdural hematoma under endoscopic evacuation. **(A)** Preoperative CT shows the subdural hematoma separation. **(B)** The skin incision is located at the coronal suture, with a backward ‘C’ shape. **(C)** The skin is open after incision. **(D)** After the valve is formed, the dura mater is cut in a cross shape. **(E)** The S shape of the suction device is used to push the brain tissue during the operation to expose the hematoma distant from the bone window. **(F)** The separation is cut. **(G)** Postoperative CT examination shows that the hematoma was removed satisfactorily. **(H)** The position, size (about 2–3 cm), and shape of the actual bone window is shown.

**Figure 7:**
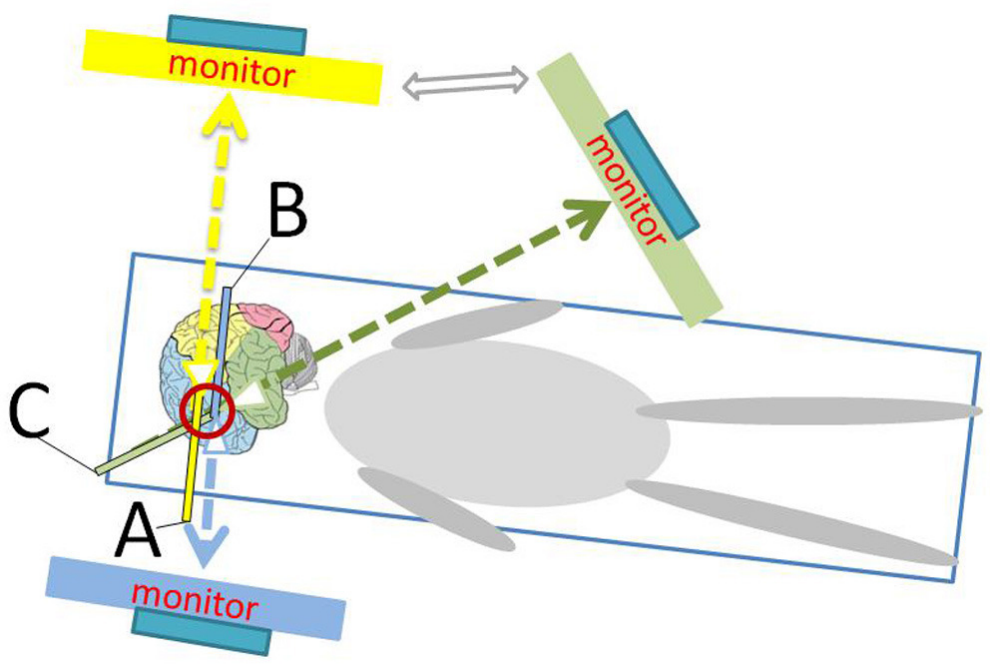
Schematic diagram shows the position of the monitor and the operator during endoscopic surgery. **(A)** represents the head of the neuroendoscope facing the parietal and occipital directions; **(B)** represents the head of the neuroendoscope facing the frontal direction; and **(C)** represents the head of the neuroendoscope facing the temporal direction.

For neuroendoscopic treatment, following general anesthesia, the head was tilted to one side at a 30–45 degrees angle and a conventional disinfection drape was set. A C-shaped incision in the frontal region was made based on the location of the hematoma indicated on preoperative imaging. The length of the surgical incision was ~9 cm; the musculocutaneous flap was turned back, and the bone flap was located on the coronal suture (**Figures 4B**, **6B**). A single hole was drilled, and a free bone flap was created. It should be noted that the inner table of the bone flap needed to be milled obliquely, and a flask-shaped bone window with a small outer diameter and a large inner diameter (Figures 1, **6H**) was created. If necessary, the bone window required further special treatment to account for the special requirements of the neuroendoscope. The inner table of the skull needed to be milled obliquely; therefore, a section of it was removed using a drill (Figures 1, 2, **6H**). The dura mater was sutured to the periosteum to prevent epidural hematoma, and a cross incision was made (**Figure 6D**). In cases where the hematoma was not visible, the capsules were cut further until the hematoma and its cavity were revealed. Next, the hematoma under the bone window (**Figure 4C**) was removed; this facilitated the introduction of the neuroendoscope into the subdural space. Suction was applied using an S-shaped curved aspirator prior to the subdural hematoma evacuation (Figure 3). The neuroendoscope was held with one hand, and an S-shaped aspirator was operated with the other hand, at various positions of the hematoma cavity (Figures 4D–F). During the operation, the arc of an S-shaped aspirator was used to push the brain tissue away to reveal and remove the hematoma that was located distant from the bone window (Figures 4F, **6E**). Effective hemostasis was necessary during surgery; therefore, bipolar cauterization was required. If the angle of bipolar insection was not convenient, the location of the bleeding could not be reached safely; therefore, the tip of the bipolar forceps was bent backward with a rongeur (Figure 5). The intraoperative fibrous septum was cut using sharp scissors (Figure 6F). The neuroendoscope and aspirator were inserted into the corresponding position for the evacuation of residual hematoma in the temporal or forehead region. If necessary, another monitor was placed in the direction of the lower limbs or forehead of patient (Figure 7) to facilitate hematoma evacuation in the temporal or forehead region. In addition, the bipolar tip was shaped to facilitate intraoperative hemostasis. Following hematoma removal, the visceral layer of the subdural hematoma remained intact without any treatment. After the neuroendoscope was withdrawn at the end of the surgery, the subdural space was filled with saline. After the dura was closed tightly, a small syringe and needle were used to fill the intracranial cavity with normal saline to avoid gas accumulation, and a drainage tube was placed under the dura mater to prevent intracranial hematoma formation. Once the dura mater was closely sutured, the bone flap was repositioned and fixed, and the drainage tube was fixed beside the incision. Finally, the surgical incision was closed layer by layer.

### Postoperative Treatment

Following the surgery, the patient was placed in the supine position, the head was inclined to the operative side, and fluid replacement was increased to promote brain tissue reduction. Epilepsy was prevented and hemostatic treatment was performed. Perioperative antibiotics were used within 24 h to prevent infection in the elderly. A head CT scan was performed within 24 h following the operation to determine whether the subdural hematoma was cleared and to check for the presence of an intracranial hematoma. The patient's consciousness, pupils, vital signs, and the amount and properties of the drainage fluid were closely observed. If the patient was conscious, their vital signs were stable on the first day following the operation, and a CT scan indicated that the subdural hematoma was cleared with the absence of any new intracranial hematoma, the drainage tube was removed. If a CT scan indicated an intracranial hematoma, the drainage continued. If necessary, urokinase was placed into the drainage tube to dissolve any intracranial blood clots, and the drainage tube was removed within 72 h post-surgery. Neurological function assessment, brain CT reexamination, and MRI examination were performed 6 months after discharge.

### Statistical Analysis

Statistical analyses were performed using SPSS for Windows (version 26.0; IBM Corp., Armonk, NY, USA). The Kolmogorov–Smirnov test was used to determine whether the data had a normal distribution. Student's *t*-test or a one-way ANOVA was used to determine significant differences in continuous data. The chi-squared test (χ^2^ test) or Fisher's exact test was used to determine significant differences in qualitative data.

## Results

A total of 43 patients with sCSDH were enrolled in our study (37 men and six women). They were categorized into neuroendoscopic treatment and standard (large bone flap) craniotomy groups, with 23 and 20 patients, respectively. The ages of the patients were 40–86 years (median: 65 years) in the standard (large bone flap) craniotomy group, and 39–84 years (median: 66 years) in the neuroendoscopic treatment group. There were no differences in sex, age, smoking, drinking, medical history, antiplatelet drug use, postoperative complications, and sCSDH recurrence between the two groups (*p* > 0.05). However, the patients in neuroendoscopic treatment group had a shorter total hospital stay and postoperative hospital stay (total hospital stay: 5.26 ± 1.89 vs. 8.15 ± 1.04 days, *p* = 0.000; postoperative hospital stay: 4.47 ± 1.95 vs. 7.96 ± 0.97 days, *p* = 0.000). The imaging and Modified Rankin Scale (mRS) at the 6-month follow-up were satisfactory, and no sCSDH recurrence was reported in the two groups.

No deaths were reported in the present study. The clinical symptoms of all our patients gradually improved within the first week after surgery, and the neurological function returned to normal within 6 months. The subdural hematomas were cleared in 42 patients; however, there was a single case with a new subdural hematoma in the surgical cavity following neuroendoscopic treatment and standard craniotomy (4.35 vs. 5.00%, *p* = 0.919). Finally, the patient was discharged after active drainage using urokinase, which was used to dissolve intracranial blood clots. There was one case (4.35%) of intracranial infection after neuroendoscopic treatment, and three (15.0%) after standard craniotomy (*p* = 0.230). There were no cases of wound infections in the neuroendoscopic treatment group and two (10.0%) in the standard craniotomy group (*p* = 0.120). While there were no cases of postoperative epilepsy in the neuroendoscopic treatment group, there were two (10.0%) such cases in the standard craniotomy group (*p* = 0.120). A single patient (4.35%) developed pulmonary infection in the neuroendoscopic treatment group, whereas there were three (15.0%) such patients in the standard craniotomy group (*p* = 0.230). All patients with postoperative complications were discharged following treatment without any complications (Table 1).

**Table 1:** Characteristics of the septated chronic subdural hematomas among the patients.

	**Standard craniotomy group (*n* = 20)**	**Neuroendoscopic treatment group (*n* = 23)**	***P*-value**
Sex			
Male	16 (80.0%)	21 (91.30%)	0.286
Female	4 (20.0%)	2 (8.70%)	
Age			
Median age (years)	65	66	0.791
Age range (years)	40–86	39–84	
Smoking	3 (15.0%)	2 (8.70%)	0.520
Drink	4 (20.0%)	6 (26.07%)	0.637
Medical history			
Hypertension	3 (15.0%)	6 (26.07%)	0.373
Diabetes	3 (15.0%)	4 (17.39%)	0.832
Cerebral infarction	2 (10.0%)	4 (17.39%)	0.485
Trauma	11 (55.0%)	9 (39.13%)	0.298
Coronary heart disease	0 (0%)	1 (4.35%)	0.345
Antiplatelet drugs	3 (15.0%)	2 (8.70%)	0.520
Postoperative complication			
Intracranial hematoma	1 (5.0%)	1 (4.35%)	0.919
Intracranial infections	3 (15.0%)	1 (4.35%)	0.230
Wound infections	2 (10.0%)	0 (0%)	0.120
Epilepsy	2 (10.0%)	0 (0%)	0.120
Pulmonary infection	3 (15.0%)	1 (4.35%)	0.230
CSDH recurrence	0 (0%)	0 (0%)	1.000
Length of total hospital stay (days)	8.15 ± 1.04	5.26 ± 1.89	0.000
Length of postoperative hospital stay (days)	7.96 ± 0.97	4.47 ± 1.95	0.000
Preoperative mRS			
0–2	19 (95.0%)	23 (100.0%)	0.278
3–6	1 (5.0%)	0 (0%)	
mRS at 6-month follow-up			
0–2	20 (100.0%)	23 (100.0%)	1.000
3–6	0 (0%)	0 (0%)	

*Values shown are n (%) or the mean ± SD. CSDH, chronic subdural hematoma; mRS, Modified Rankin Scale*.

## Discussion

Neuroendoscopic treatment is a potential therapeutic strategy for the treatment of sCSDH, but the safety and efficacy of neuroendoscopic treatments remain controversial. For symptomatic patients with CSDH, burr hole evacuation is often the first-line treatment choice (5). However, treatment of sCSDH using burr hole evacuation is reported to be associated with a high rate (7–10%) of hematoma recurrence (11, 12) or residual hematoma and, therefore, requires further surgical intervention (7). While standard (large bone flap) craniotomy is an efficacious treatment in sCSDH, it requires a longer incision and a longer operating time, has high rates of postoperative intracranial infections, wound infections, epilepsy, and requires a relatively longer duration in the hospital (2, 13–15); these are not consistent with the current trend of minimally invasive surgery. Our study showed that there were no differences in sex, age, smoking, drinking, medical history, taking antiplatelet drugs, postoperative complications, and sCSDH recurrence between the neuroendoscopic treatment and standard craniotomy groups (*p* > 0.05). However, patients in the neuroendoscopic treatment group had a shorter length of total hospital stay and postoperative hospital stay than those in the standard craniotomy group. Furthermore, the imaging and mRS at the 6-months follow-up were satisfactory, with no reports of sCSDH recurrence. Therefore, sCSDH can be safely and effectively treated using neuroendoscopic treatment.

With advancements in microscopy and neuroendoscopic techniques, the treatment of patients with sCSDH has gradually become minimally invasive. None of the 23 patients in the neuroendoscopic treatment group developed wound infections or epilepsy. Our study showed that there were no differences in postoperative complications and sCSDH recurrence between the neuroendoscopic treatment and standard craniotomy treatment. Patients with limb weakness in the neuroendoscopic treatment group were able to perform morning bedside early rehabilitation measures, which helped to ease wound pain and prevent complications. This led to the patients in the neuroendoscopic treatment group having a shorter total hospital stay and postoperative hospital stay (*p* = 0.000).

Considering its efficacy, we believe that the neuroendoscopic treatment technique must be mastered by other clinical centers. Here, we summarized, analyzed, and compared the clinical data of 43 patients with sCSDH treated with neuroendoscopic treatment or standard (large bone flap) craniotomy. The surgical procedures for neuroendoscopic treatment are described in detail. First, we focused on making the incision as small and minimally invasive as possible. The frontal C-shaped incision was moved backward, making more space for the endoscope to be closer to the skull, so that the subdural tip of the neuroendoscope could be operated away from the brain tissue, thereby facilitating operating the neuroendoscope from the forehead to the back. Based on the minimally invasive incision, we improved the formation of the bone window, milling, and cutting of the skull into a flask-shaped bone flap, with a smaller outside surface and a larger inside surface, that facilitates entry and exit for neuroendoscopy. In addition, we bent the suction device into an S shape that allowed the upturned tip to be positioned away from the cerebral cortex and be used to push the brain tissue away to facilitate the removal of the remnant hematoma. In addition, it was necessary to shape the tip of the bipolar forceps for hemostasis of the dura mater and fibrous septum.

Our study had some limitations. This was a retrospective study, it was limited by the number of cases, and the groups we analyzed did not include a multiple burr hole group or orally medicated Lipitor group. These limitations should be addressed in future clinical research on the endoscopic treatment of patients with sCSDH.

In conclusion, our study showed that neuroendoscopic evacuation is an effective and safe treatment for sCSDH, it is minimally invasive, and it should be utilized clinically. However, it should be noted that there were only 23 cases in this study, and the results of this study should be verified using clinical trials that include a larger number of patients.

## Data Availability Statement

The original contributions presented in the study are included in the article/supplementary material, further inquiries can be directed to the corresponding author/s.

## Ethics Statement

The studies involving human participants were reviewed and approved by the Ethics Committee of the First Affiliated Hospital of Fujian Medical University (no,: MRCTA, ECFAH of FMU [2019] 123). Written informed consent for participation was not required for this study in accordance with the national legislation and the institutional requirements.

## Author Contributions

JD, MZ, HW, GC, and HS: acquisition of data and critical revision of manuscript for intellectual content. LY and CJ: study supervision. WF, FW, and PY: study concept and design. DK and SZ: analysis and interpretation of data and study supervision. All authors reviewed the manuscript.

## Funding

This study was supported by the Fujian Clinical Research Center for Neurological Disease (SSJ-YJZX-1 to DK), Key Clinical Specialty Discipline Construction Program of Fujian, P.R.C, a major project of Fujian Provincial Department of Science and Technology (Nos. 2014YZ0003 and 2014YZ01 to DK), Young and Middle-aged Backbone Key Research Project of National Health and Family Planning Commission of Fujian Province (No. 2017-ZQN-46 to PY), Natural Science Funding of Fujian Province (Nos. 2018J01175 to PY and 2018J01176 to SZ), and Natural Science Funding of China (No. 81802492 to PY).

## Conflict of Interest

The authors declare that the research was conducted in the absence of any commercial or financial relationships that could be construed as a potential conflict of interest.

## Publisher's Note

All claims expressed in this article are solely those of the authors and do not necessarily represent those of their affiliated organizations, or those of the publisher, the editors and the reviewers. Any product that may be evaluated in this article, or claim that may be made by its manufacturer, is not guaranteed or endorsed by the publisher.
